# The Depression Anxiety Stress Scale-21 in Chinese Hospital Workers: Reliability, Latent Structure, and Measurement Invariance Across Genders

**DOI:** 10.3389/fpsyg.2020.00247

**Published:** 2020-03-06

**Authors:** Li-chen Jiang, Ya-jun Yan, Zhi-Shuai Jin, Mu-Li Hu, Ling Wang, Yu Song, Na-Ni Li, Jun Su, Da-Xing Wu, Tao Xiao

**Affiliations:** ^1^Medical Psychological Center, The Second Xiangya Hospital, Central South University, Changsha, China; ^2^Research Office, The Second Xiangya Hospital, Central South University, Changsha, China; ^3^Department of Rehabilitation, Hunan Provincial People’s Hospital, The First Affiliated Hospital of Hunan Normal University, Changsha, China; ^4^Department of Science and Education, Zhuzhou 331 Hospital, Zhuzhou, China

**Keywords:** depression anxiety stress scale-21, reliability, latent structure measurement invariance, Chinese version, gender

## Abstract

The Depression Anxiety Stress Scale-21 (DASS-21) is an instrument in the assessment of mental health status. The current study recruited 1,532 Chinese hospital workers [74.4% female; mean age = 31.97 (SD = 9.70) years] to examine the reliability, latent structure, and measurement invariance of the DASS-21 between genders. The Cronbach’s α values were greater than 0.90 for total score. This study examined four possible models of the DASS-21 using the confirmatory factor analysis (CFA) in Chinese hospital workers. The results from CFA revealed that the latent structure of the DASS-21 in medical staffs is best represented by a one-factor model. Then we used the one-factor model to examine measurement invariance across genders by using a multiple-group categorical CFA. All values of root mean square error approximation (RMSEA) were less than 0.08, all Comparative Fix Index (CFI) and Tucker–Lewis Index values were greater than 0.90, all ΔCFI (changes in CFI) values were less than 0.010, and ΔRMSEA (the changes in RMSEA) were less than 0.015. These findings supported the gender invariance of the DASS-21 among Chinese hospital workers.

## Introduction

The Depression Anxiety Stress Scale (DASS) is a widely used instrument developed by Lovibond and Lovibond (1995b) to measure anxiety, depression, and stress. This self-reported questionnaire has 42 items. Seven items with the highest loadings from each subscale of the original DASS were selected to develop the DASS-21. The DASS was originally intended to consist of only two subscales, depression and anxiety. It covered all the core symptoms of depression and anxiety and provided the biggest discrimination between the subscales. However, during scale development, the stress subscale emerged, and more items were added to the subscale. Lovibond and Lovibond (1995b) label it “Stress” or “Tension.” The original three-factor model of Lovibond and Lovibond (1995a) includes the three factors of depression, anxiety, and stress (same as the three subscales of the DASS-21). The depression subscale is characterized by hopelessness, self-deprecation, low positive affect, and devaluation of life; the anxiety subscale is related to physiological hyperstimulation and a subjective consciousness of anxious affect, and the stress subscale is a collection of items such as relaxation difficulties, tension, impatience, irritability, and restlessness. Therefore, the depression, anxiety, and stress subscales have common characteristics, including negative affect, emotional distress, and changes in physiology in the hypothalamic–pituitary–adrenal axis (Mello et al., 2007).

Prior criticism regarding the psychometric properties of the DASS-21 has centered on its latent construct (e.g., Brown et al., 1997; Gong et al., 2010; Henry and Crawford, 2005; Le et al., 2017, although some researchers believed that this scale embodies a special, independent emotional syndrome. For example, the original three-factor model raised by Lovibond and Lovibond (1995a) and the tripartite three-factor model. The tripartite three-factor model of the DASS-21 was in accord with Watson and Clark’s (1984) tripartite conceptualization. It includes three factors – physiological hyperarousal, anhedonia, and generalized negativity. The tripartite model proposed by Watson and Clark (1984) includes three parts – a depression-type factor (characterized by low positive affect), a physiological hyperarousal factor (includes some side of anxiety), and a negative affectivity factor (contains the common mood states associated with depression and anxiety). Watson and Clark (1984) argued that the potential existence of negative affectivity could explain the high correlation in the study of mood disorders. However, negative emotion is a vague concept that is generally considered to include several aspects that are difficult to distinguish. There is also evidence that the DASS-21 is mainly related to the composition of general negative emotions. For example, Brown et al. (1997) argued that scores of the DASS-21 were primarily related to the construct of general negative affect. Duffy et al. (2005) used confirmatory factor analysis (CFA) and found that the results did not support the original three-factor structure and the tripartite three-factor model in children aged 11 to 15 years. Instead, a two-factor model consisting of “physiological hyperarousal” and “general negative affect” can fully fit the data after correlating the error terms of two items. Furthermore, both Tran et al. (2013) and Camacho et al. (2016) identified a one-factor solution in non-clinical samples by using exploratory factor analysis (EFA) (*N* = 221 and *N* = 505, respectively), and Camacho et al. (2016) suggested that the items of the DASS-21 were best represented by one factor, namely, anxious/stress–depression factor. Thus, we aim to explore the latent structure of the DASS-21 in Chinese hospital workers.

Until now, the DASS has been translated into 50 languages^1^, making it widely accessible to researchers across the globe. In the last two decades, several studies in Western culture have supported the psychometric characteristics and utility of the original English version of the DASS-21, including the United States, Spain, and the United Kingdom (e.g., Brown et al., 1997; Daza et al., 2002; Crawford and Henry, 2003), and recently to Eastern culture, including China and Japan^1^. Taouk et al. (2001) reported the DASS-21 for Chinese speakers in Hong Kong to use the traditional Chinese character set. Zuo and Chang (2008) directly translated the DASS from the original English version into simplified Chinese characters. However, back-translation from simplified Chinese to English was not performed, and preservation of item meaning has not been confirmed [Zuo et al. (2012), personal communication, April 29, 2012]. While the Chinese version of the DASS-21 has proven useful for field work, there are currently few data to support the psychometric characteristics of this test used in China (Chan et al., 2012). In addition, other studies have shown that the effectiveness of psychological measurement instruments may differ among populations because of differences in cultural, language expression, and values (Gjersing et al., 2010). Furthermore, there is a lack of validation studies among Asian samples on cultural differences that may make significant variations (Oei et al., 2013). For example, depression, anxiety, and stress measured by DASS-21 are considered to be different in east and west culture (Mellor et al., 2015). Marsella et al. (1985) reported that in Western populations symptoms of depression are dominated by sadness and feelings of worthlessness, whereas in non-Western populations, somatic symptoms such as sleep difficulties predominate. Leong et al. (2003) suggested that there were some cross-cultural differences in the average scores on the depression and anxiety tests. For example, some studies (e.g., Baron and Matsuyama, 1988; Shek, 1991; Chan, 1995) reported that Asian adolescents and adults have higher average scores of various depression scales than the average score previously reported for their peers in North America in the original version of the same instrument. Many studies in China also have shown that the average score of patients and non-patients on various Chinese depression scales is higher than the average score of their American counterparts (e.g., Chan and Tsoi, 1984; Chan, 1991). Also, about the DASS, Norton (2007) found that when exploring the relationship between the depression subscale and negative emotions, a small race effect was observed. When examining the relationship between the depression subscale and Beck Depression Inventory scores, it was found that African–American participants were stronger than Asian, Caucasian, or Hispanic participants. In terms of the relationship between the depression subscale and the PANAS-NA (the Negative Affect dimensions of Positive Affect Negative Affect Schedule), Caucasian and Hispanic participants were significantly stronger than African–American or Asian participants. Therefore, what the DASS-21 measures may not be exactly the same in different cultures, so it is necessary to continue to study the simplified Chinese version of the DASS-21.

The DASS also has been widely used in a variety of settings since its release in 1995, including clinical and non-clinical groups (Antony et al., 1998; Crawford and Henry, 2003; Henry and Crawford, 2005), in a variety of countries (Taouk et al., 2001; Gong et al., 2010; Chan et al., 2012), and among different age groups (Szabó, 2010; Osman et al., 2014). However, there is little research on the use of the DASS-21 among medical professionals. Lee et al. (2011) studied the internal consistency of the DASS on Korean hospital workers, but they did not study other important psychometric characteristics of the DASS-21. According to previous research, medical staff in many countries, including China, are liable to feel high levels of depression, anxiety, and stress (e.g., Caplan, 1994; Fagnani Neto et al., 2004; Ahmed et al., 2009). These studies have shown that the level of psychological distress of medical professionals is higher than that of the general public (Zhou et al., 2018). In China, Xu and Zhao (2006) reported that 28% of general hospital doctors have psychological problems, and mental health was significantly worse than that in normal adults. Researches have shown that 47.2% of medical staff suffer from depression. The mental health status of medical staff directly affects the quality of medical service and patient safety. Ignoring the mental health status of medical professionals will negatively affect the quality of medical treatment (Zhou et al., 2018). Therefore, it is necessary to ascertain the psychometric properties of the DASS-21 among Chinese medical staffs, which is conducive to its application in medical staff and has an important role in evaluating the negative emotions of medical staffs.

Differences in experiences of distress and coping mechanisms have been found between genders. Many studies have shown that females are significantly more likely to suffer from anxiety and depression disorders than males (Bruce et al., 2005; Song et al., 2008). Grant and Weissman (2007) suggested that there are gender differences in interpretation, recall, and self-reporting of afflict inventory items, and women are more likely to respond more severely to self-reporting lists (Ritvo et al., 2008). Many studies have explored gender differences on the DASS-21 total score and depression, anxiety, and stress subscales, but the results have been inconsistent. For example, Szabó (2010) reported that girls (mean age = 13.62 years) experienced significantly higher levels of depression and stress than boys. Crawford and Henry (2003) reported that women scored significantly higher than did men on the anxiety scale, depression scale, and total of the three scales, but the gender difference on the stress subscale was not statistically significant in the sample of United Kingdom adults (mean age = 40.9 years). Gong et al. (2010) found that the score on the depression subscale of boys was significantly higher than that of girls in the sample of college students (mean age = 19 years). It can be found that in different samples the gender difference results of the DASS-21 total scale and each subscale are inconsistent. Currently, the DASS-21 scoring system is the same for both men and women, but cross-group measurement invariance is necessary when using the same instrument across different groups (Reise et al., 1993). If we do not evaluate measurement invariance, the comparison of means between male and female medical stuffs could be problematic. A number of studies (Wu and Huang, 2014; Doi et al., 2018) have demonstrated that other depression and anxiety measurement scales have reliable measurement invariance (e.g., Beck Depression Inventory-II, the 7-item Generalized Anxiety Disorder Scale). Some studies have examined the measurement invariance of the DASS-21 across genders (Gomez et al., 2014; Jafari et al., 2017; Lu et al., 2018). Gomez et al. (2014) tested the measurement invariance of the DASS-21 across ratings provided by men (*N* = 227) and women (*N* = 460) in an American community sample. These authors treated item-level data as continuous and used a maximum likelihood estimation. However, the DASS-21 items are more appropriately scored on a 4-point Likert scale, and the weighted least-squares method with mean and variance adjusted (WLSMV) is used to more accurately treat the data as categorical data (Lu et al., 2018). Employing a categorical CFA (CCFA), Jafari et al. (2017) found that the DASS-21 is gender-based measurement invariance among medical students, although the psychological distress of medical students is higher than the general population. In addition, using WLSMV to analyze the data of the DASS-21, Lu et al. (2018) found support for gender-based measurement invariance in a Chinese sample. However, the samples of this study are students mainly from normal universities. To date, there have been no similar studies examining the psychometric properties of the DASS or DASS-21 in hospital workers. It is necessary to investigate the psychometric properties of the Chinese version of the DASS-21 within hospital workers to ensure that the instrument can be used as the original instrument (Scholten et al., 2017).

The current study aimed to determine the reliability and to evaluate competing models of the latent structure of the DASS-21 among a sample of Chinese hospital workers. In addition, we aimed to extend previous research by using multiple-group CFA to validate the measurement invariance of the DASS-21 across genders in Chinese hospital workers.

## Materials and Methods

### Participants

Of the 1,575 medical personnel from three hospitals in China asked to participate in the study, 1,532 volunteered (response rate of 97%). Of the 1,532 subjects, 472 were doctors (30.6%), 812 were nurses (52.6%), 107 were medical technicians (6.9%), 137 were administrative staff (8.9%), and 12 were categorized as “other” (0.8%). Women make up the larger portion of the sample (74.4%). In the female sample, 18.7% were doctors, 67.4% were nurses, and 5.3% were medical technicians, and 8.7% were administrative and other personnel. In the male sample, 66.2% were doctors, 9.4% were nurses, 12% were medical technicians, and 12.3% were administrative and other personnel. The average age of participants was 31.97 (SD = 9.70) years (range = 19–73 years). The average number of years working in the medical field was 9.70 (SD = 10.38) years (range = 0–55 years).

### Instruments

The self-report questionnaire consists of two sections and takes approximately 10 min to complete. The first section collected data on sociodemographics including gender, age, marital status, profession, years working in the medical field, and years of education. The second section measured depression, anxiety, and stress as assessed by the DASS-21 translated by Yi et al. (2012). This 21-item scale is easy to apply in both clinical and non-clinical settings and is used to measure the negative emotions of individuals in the most recent week. Each subscale contains seven items. Participants were asked to respond on how closely the item applied to them in the past week. The scale uses the Likert four-level scoring system, with 0 to 3 points representing non-conformity (0) to very consistent (3). The higher the score, the higher the level of negative emotions.

### Statistical Analyses

Data were analyzed with SPSS 20.0 (SPSS, 2011) and Mplus 7.0 (Muthén and Muthén, 1998-2012). The reliability of the DASS-21 was analyzed with SPSS 20.0. The Cronbach’s α coefficient (reliability) of the DASS-21 was considered adequate (α > 0.70). Independent-samples *t* tests were used to test gender difference of the total scale and three subscales. We used CFA to evaluate competing models of the latent structure of the DASS-21 by Mplus 7.0, and then the best-fit model was used to evaluate the measurement invariance. Given the response scale of the DASS-21, indicator variables were treated as categorical rather than continuous, and therefore the WLSMV estimator and theta parameterization were used in all CFAs. According to Hu and Bentler (1999), Comparative Fix Index (CFI) and the Tucker–Lewis Index value of 0.90 indicate adequate fit (and >0.95 indicates excellent fit). A root mean square error approximation (RMSEA) value of less than 0.08 indicates good fit. We used the fit indices of each model and the ΔCFI (the changes in CFI) between each model and the other three competing models to compare models. If the difference in the ΔCFI between two competing models is less than —0.01—, then we should not reject the hypothesis that no difference in fit between the two models and the more parsimonious model should be retained (Cheung and Rensvold, 2002). The four competition factor models include the original three-factor model, the tripartite three-factor model, the two-factor model (Duffy et al., 2005), and the one-factor model. The original three-factor structure includes three factors of depression, anxiety, and stress, and the tripartite three-factor model contains factors of physiological hyperarousal, anhedonia, and generalized negativity. Two-factor models concludes a general negative affect factor and a physiological hyperarousal factor, and one-factor model that all items are loading on a general factor.

To examine the measurement invariance across genders of the DASS-21, we used a multiple-group CFA in Mplus 7.0. For each comparison, three models were evaluated: M1. Configural invariance: same underlying factor structure across groups, established before conducting measurement invariance test; M2. Weak or metric invariance: invariant factor loadings across groups for gender invariance while thresholds were free to vary; M3. Strong or scalar invariance: factor loadings and thresholds are equal across groups (Dimitrov, 2010). First, we investigated whether the hypothesized one-factor model fits the data for each gender group as well as the whole sample. Second, we figured out a configural invariance model (M1). Then we used the nested model 2 (M2) to test the metric (weak) invariance, where factor loadings were equal between different genders. Finally, we tested the scalar (strong) invariance with nested model 3 (M3), where the loadings and thresholds were limited to be equal between the male and female groups (Van de Schoot et al., 2012). When M2 and/or M3 match the data as well as M1, the metric and/or scalar invariance was indicated. The establishment of both forms of invariance indicated the DASS-21 has meaningful comparability across genders (Millsap, 1998). We used the DIFTEST option in Mplus for model comparisons. It is well known that the χ^2^ test is too sensitive to the measurement-invariance evaluation in large samples (*N* > 300) (Chen, 2007), and therefore, we used the ΔCFI and ΔRMSEA between the comparison and nested models to evaluate measurement invariance. The measurement invariance was indicated by ΔCFI < 0.010 and ΔRMSEA < 0.015 as described by Chen (2007) and Cheung and Rensvold (2002).

## Results

### Summary Statistics and Reliability of the DASS-21

Table 1 lists statistics of the DASS-21 for the whole sample, male and female groups. The Cronbach’s α coefficients of the total scale of the DASS-21 were calculated for internal consistency. The Cronbach’s α coefficient was 0.95 for the total DASS-21 scale. Independent-samples *t* tests revealed that men obtained significantly higher scores than did women on the depression subscale and total of the three subscales. The gender difference on the anxiety and stress subscale did not achieve statistical significance.

**TABLE 1:** Summary statistics for the DASS-21 among 1,532 hospital workers in China.

	**Male(*n* = 383)**			**Female(*n* = 1,149)**			***t*(*p*)**
	**Mean**	**SD**	**Range**	**Mean**	**SD**	**Range**	
Depression	7.24	8.089	42	5.90	7.025	36	2.891**(0.004)
Anxiety	6.70	7.775	42	6.08	6.392	38	1.425 (0.155)
Stress	9.27	8.349	42	8.49	7.243	36	1.645 (0.100)
Total scale	23.22	23.064	126	20.47	19.239	104	2.101*(0.036)

Scores on the DASS-21 are multiplied by 2 to calculate the final subscale scores, producing a maximum of 42 points. * p < 0.05, ** p < 0.01.

### Factor Structures

The WLSMV was used to perform CFA to evaluate four models of the latent structure of the DASS-21. First, we tested the original three-factor structure suggested by Lovibond and Lovibond (1995a). This model provides a satisfactory fit, but the correlation between depression factor and anxiety factor (*r* = 0.933), depression factor and stress factor (*r* = 0.951), and depression factor and stress factor (*r* = 0.951) suggested indistinguishability of these factors (Table 2). Next, the tripartite three-factor model developed from Watson and Clark (1984) was tested. The data fit the model well, and there were also strong interfactor correlations between the three factors (physiological hyperarousal–anhedonia = 0.894, physiological hyperarousal–generalized negativity = 0.928, anhedonia–generalized negativity = 0.980), suggesting these factors were also not distinguishable. Then, Duffy et al. (2005) two-factor models were evaluated. The fit indices for this model were receivable, and the correlation coefficient of the two factors is also high (*r* = 0.921). This indicates that the physiological hyperarousal and the general negative affect factors do not appear to be independent constructs. Finally, we tested the one-factor model. As Table 2 shows, all models provide an adequate fit. These results indicate that all fit indices exceeded accepted values suggested by Hu and Bentler (1999), but the interfactor correlations of the three- and two-factor models are too high, and all the ΔCFI values between competing models are smaller than —0.01—. Therefore, this study uses the one-factor model for further research. Table 3 provides the factor loadings for the one-factor model of the male and female groups. Overall, all item loadings are significant (*p* < 0.001) and high, ranging from 0.603 to 0.921.

**TABLE 2:** Model fit indices for the four models tested.

**Model**	**χ^2^ (df)**	**CFI**	**TLI**	**RMSEA (90% CI)**
The original three-factor model	1,792.196 (186)	0.969	0.965	0.075 (0.072–0.078)
The tripartite three-factor model	1,815.892 (186)	0.968	0.964	0.076 (0.072–0.079)
Two-factor model	1,874.961 (188)	0.967	0.963	0.076 (0.073–0.079)
One-factor model	1,984.936 (189)	0.965	0.961	0.078 (0.075–0.082)

*df, degree of freedom; TLI, Tucker–Lewis Index; CFI, Comparative Fit Index; RMSEA, root mean square error of approximation; 90% CI, 90% confidence interval for RMSEA.*

**TABLE 3:** Stand loading of each item of the DASS-21 across genders.

**Subscale, (item number), and item name**	**Std. loading**	
	**Male**	**Female**
**Depression subscale**		
(3) Positive feelings	0.833	0.810
(5) Initiative	0.764	0.725
(10) Look forward to	0.867	0.866
(13) Down-hearted and blue	0.911	0.885
(16) Enthusiastic	0.833	0.778
(17) Self-worth	0.911	0.817
(21) Life was meaningless	0.909	0.859
**Anxiety subscale**		
(2) Dryness of mouth	0.773	0.603
(4) Breathing difficulty	0.850	0.751
(7) Trembling	0.782	0.668
(9) Worry about panic and making a fool of self	0.807	0.714
(15) Close to panic	0.899	0.873
(19) Action of my heart	0.852	0.773
(20) Scared	0.877	0.799
**Stress subscale**		
(1) Wind down	0.730	0.682
(6) Overreact	0.805	0.696
(8) Nervous energy	0.827	0.775
(11) Agitated	0.921	0.895
(12) Difficult to relax	0.870	0.882
(14) Intolerant	0.750	0.685
(18) Touchy	0.785	0.709

*All item loadings are significant (*p* < 0.001).*

### Measurement Invariance Between Genders

Subsequently, we tested measurement invariance of the one-factor model across the male and female groups. First, single-group CFAs were performed to check the structural validity of the DASS-21 in each gender group (see Table 4, Model C in the male/female groups). Within each gender, the one-factor model demonstrated acceptable fit. The configural invariance test was performed by studying a baseline model without constrained parameter across the two genders (M1). This model fit the data well. Thus, we tested the metric invariance model. The metric invariance model (M2) was acceptable in all fit indices. Moreover, the ΔCFI and ΔRMSEA (M1 vs. M2) were less than 0.010, supporting metric invariance and suggesting that factor loadings were invariant across genders. Given these results, we continued to test scalar invariance. This model (M3) showed receivable fit. Moreover, the ΔCFI and ΔRMSEA (M2 vs. M3) values were also within recommended ranges, supporting the scalar invariance and suggesting that factor loadings and item thresholds were invariant across genders. These results indicate that item thresholds on the latent constructs were invariant across genders. In summary, the results of multiple-group CFAs supported configural invariance, metric invariance, and scalar invariance of the DASS-21, revealing that the factor structure, factor loadings, and item thresholds were fully equivalent across genders.

**TABLE 4:** Results of confirmatory factor analysis and measurement invariance across genders.

**Model**	**χ^2^ (df)**	**CFI**	**TLI**	**RMSEA (90%CI)**	**Model comparison**	**ΔRMSEA**	**ΔCFI**
C in total sample (*n* = 1,532)	1,984.936(189)	0.965	0.961	0.078 (0.075–0.082)	–	–	–
C in male group (*n* = 383)	526.520 (189)	0.982	0.980	0.068 (0.061–0.075)	–	–	–
C in female group (*n* = 1,149)	1,527.880(189)	0.965	0.961	0.079 (0.075–0.082)	–	–	–
1.Configural invariance (M1)	1,912.469(378)	0.972	0.968	0.073 (0.070–0.076)	–	–	–
2.Metric invariance (M2)	1,949.453(398)	0.971	0.970	0.071 (0.068–0.075)	2 vs. 1	0.001	0.002
3.Scalar invariance (M3)	1,770.140(439)	0.975	0.976	0.063 (0.060–0.063)	3 vs. 2	0.004	0.008

*ΔCFI and ΔRMSEA refer to the difference between the model under consideration and the preceding (less constrained) model. df, degree of freedom; TLI, Tucker–Lewis Index; CFI, comparative fit index; RMSEA, root mean square error of approximation; 90% CI, 90% Confidence Interval for RMSEA; ΔCFI, difference in CFI between nested models; ΔRMSEA, difference in RMSEA between nested Models.*

## Discussion

### Summary Statistics and Reliability of the DASS-21

One of the purposes of the present study was to estimate psychometric properties of the simplified Chinese version of the DASS-21 among a sample of Chinese hospital workers. Anastasi and Urbina (1997) suggested that good reliability is indicated by Cronbach’s α > 0.85. The reliability of the DASS-21 was high than this criterion (0.95), indicating that this scale is a reliable psychometric instrument with good internal consistency. Previous studies have found similar results in non-clinical and clinical groups (Antony et al., 1998), in diabetic patients (Norton, 2007), in undergraduate and graduate students (Imam, 2008), and in a young adolescent sample (Szabó, 2010). The result of the independent-samples *t* tests revealed that on the depression subscale and the total scale men scored significantly higher than did women. Most previous studies have suggested that women suffer more negative emotions (such as depression and anxiety) than do men (e.g., Hope and Henderson, 2014), but there are also a few studies that show no significant differences between men and women (e.g., Al Sunni et al., 2014). This article found that the levels of depression and negative emotions in the male group were significantly higher than those in the female group. The occupational ratio of male and female samples collected in this study may have resulted from the inconsistent consequences. In this study, nurses accounted for the majority of female samples (67%), whereas doctors accounted for the majority of the male groups (66.2%). While doctors usually work on the clinical frontline and may face more risks and conflicts, as a result, the level of depression of doctors may be more than that of nurses (Wu et al., 2010; Pang et al., 2014).

### Factor Structures of the DASS-21

The original three-factor structure of the DASS-21 has been confirmed among diverse cultural and ethnic samples (e.g., Norton, 2007). However, there also are discrepant findings about its factor structure, ranging from one-factor (Patrick et al., 2010), two-factor (Duffy et al., 2005), and three-factor (Tully et al., 2009; Szabó, 2010) to four-factor (Szabó, 2010) structures. Hence, one purpose of this study was to explore the latent structure of the DASS-21 in Chinese hospital workers. Our study found that the three factors of the DASS-21 are conceptualized considered to be either depression, anxiety and stress; or physiological hyperarousal, anhedonia, and general negative effects; or physiological hyperarousal and general negative affect; the interfactor correlations were so large that it is impossible to establish conceptual independence. Similar results were found in the following studies. Patrick et al. (2010) evaluated the underlying structure of the DASS-21 in Australian children and adolescents sample, and the results showed that the test was difficult to distinguish between depression and anxiety and stress in this sample; it seems to measure a general distress dimension. Similarly in college students, Camacho et al. (2016) identified a one-dimension factor (anxious/stress–depression) by using exploratory factor analysis (EFA). Tran et al. (2013) found that all items of the DASS-21 were loaded on one factor in the Vietnamese female sample, and they concluded that there were no subscales or combinations that could differentiate between depression and anxiety. Similar findings were reported from a study in clinical population in Egypt. Ali and Green (2019) found that a one-factor solution was most appropriate for the DASS-21 data among Egyptian drug users.

These results suggest that it is more reasonable to view the DASS-21 as measuring only a one factor of negative affectivity or general psychological distress in Chinese hospital workers. In addition, the interfactor correlations of the models reflect limited discriminant power. Hence, perhaps the DASS-21 should be used in a sample of Chinese medical staffs with caution, and it may be best to see it as a measure of undifferentiated negative affect until further certification for the discriminate validity of depression, anxiety, and stress subscales becomes available.

### Measurement Invariance Between Genders

The results of the current study sustain the measurement invariance of the DASS-21 between genders among Chinese hospital workers. The results of the single-group CFA for each gender group as well as the whole sample and configural invariance in favor of the one-factor latent structure of the DASS-21 for both males and females indicate that the DASS-21 gauged the same structures among male and female Chinese hospital workers. In addition, acceptable metric invariance indicates that the DASS-21 measures negative affective emotion in a similar manner among men and women. Furthermore, the current study found that the results of scalar invariance between genders indicate that the thresholds of each items of the DASS-21 were equal. These results suggest that comparing the scores of the DASS-21 across genders in the current sample of Chinese hospital workers is meaningful.

Because the DASS-21 scale measures negative affective emotion similarly for both females and males, it is not necessary to use different normative scores between genders. These results are consistent with the results of Gomez et al. (2014), who used an Australian sample (mean age = 47.37 years) to prove that the DASS-21 is gender-invariant, as demonstrated by Lu et al. (2018), who used a sample of Chinese college students (mean age = 19.7 years) to prove that that the DASS-21 was gender-invariant. Although the factor model used are different, Gomez et al. (2014) and Lu et al. (2018) both used the original three-factor model, and we used a one-factor model, but our current research on a sample of Chinese hospital workers reached the same conclusion, showing that application of the DASS-21 is not influenced by gender. Our findings therefore provide extra support for the DASS-21, extending prior studies in applications and research environments.

While the current study confirms the validity and reliability of the DASS-21 among genders, the study should still consider some limitations. First, half of the sample in the current study was nurses; as such, the proportion of females was significantly larger than males. While the number of males in the sample was sufficient, it should be noted that the results may not be equally representative of both genders. The second limitation of this study was the self-assessment methodology of the questionnaire, lacking clinical assessment to confirm levels of depression, anxiety, and stress. Further researches are needed to confirm latent structure and discriminate validity, sensitivity, and specificity of the DASS-21 as an effective screening instrument in various populations. Third, the sample was not recruited randomly, and thus, the interpretations of the findings are limited. Fourth, the test–retest reliability of the DASS-21 was not investigated in this study. However, as the instrument is intended to measure a state rather than trait, it would be expected that test–retest stability would be low. It would be helpful to understand the degree of stability observed when symptoms do not fluctuate.

## Data Availability Statement

The datasets generated for this study are available on request to the corresponding author.

## Ethics Statement

This study was approved by The Second Xiangya Hospital of Central South University Ethics Committee. Informed consent was obtained from all subjects.

## Author Contributions

D-XW and TX conceived and designed the study. All authors were involved in the study conduction. LJ and D-XW performed the analysis and prepared the manuscript. All co-authors contributed substantially to its revision and approved the final manuscript.

## Conflict of Interest

The authors declare that the research was conducted in the absence of any commercial or financial relationships that could be construed as a potential conflict of interest.

## Footnotes

1
www2.psy.unsw.edu.au/dass/
