# Corrigendum: The Depression Anxiety Stress Scale-21 in Chinese Hospital Workers: Reliability, Latent Structure, and Measurement Invariance Across Genders

**DOI:** 10.3389/fpsyg.2020.00741

**Published:** 2020-04-15

**Authors:** Li-chen Jiang, Ya-jun Yan, Zhi-Shuai Jin, Mu-Li Hu, Ling Wang, Yu Song, Na-Ni Li, Jun Su, Da-Xing Wu, Tao Xiao

**Affiliations:** ^1^Medical Psychological Center, The Second Xiangya Hospital, Central South University, Changsha, China; ^2^Research Office, The Second Xiangya Hospital, Central South University, Changsha, China; ^3^Department of Rehabilitation, Hunan Provincial People's Hospital, The First Affiliated Hospital of Hunan Normal University, Changsha, China; ^4^Department of Science and Education, Zhuzhou 331 Hospital, Zhuzhou, China

**Keywords:** depression anxiety stress scale-21, reliability, latent structure measurement invariance, Chinese version, gender

In the published article, the author Tao Xiao was given the wrong affiliation. The author should have affiliation 2 instead of affiliation 3.

The authors apologize for this error and state that this does not change the scientific conclusions of the article in any way. The original article has been updated.

