# Corrigendum: The Depression Anxiety Stress Scale-21 in Chinese Hospital Workers: Reliability, Latent Structure, and Measurement Invariance Across Genders

**DOI:** 10.3389/fpsyg.2022.899246

**Published:** 2022-05-31

**Authors:** Li-chen Jiang, Ya-jun Yan, Zhi-Shuai Jin, Mu-Li Hu, Ling Wang, Yu Song, Na-Ni Li, Jun Su, Da-Xing Wu, Tao Xiao

**Affiliations:** ^1^Medical Psychological Center, The Second Xiangya Hospital, Central South University, Changsha, China; ^2^Research Office, The Second Xiangya Hospital, Central South University, Changsha, China; ^3^Department of Rehabilitation, Hunan Provincial People's Hospital, The First Affiliated Hospital of Hunan Normal University, Changsha, China; ^4^Department of Science and Education, Zhuzhou 331 Hospital, Zhuzhou, China

**Keywords:** depression anxiety stress scale-21, reliability, latent structure measurement invariance, Chinese version, gender

In the original article, there was an error. In section “*Instruments*” of the original article, a reference was incorrectly written as “Zuo and Chang (2008).” Instead, it should be “Yi et al. (2012).” A correction has been made to “*Instruments*,” Paragraph 1. The corrected paragraph is shown below.

The self-report questionnaire consists of two sections and takes approximately 10 min to complete. The first section collected data on sociodemographics including gender, age, marital status, profession, years working in the medical field, and years of education. The second section measured depression, anxiety, and stress as assessed by the DASS-21 translated by Yi et al. (2012). This 21-item scale is easy to apply in both clinical and non-clinical settings and is used to measure the negative emotions of individuals in the most recent week. Each subscale contains seven items. Participants were asked to respond on how closely the item applied to them in the past week. The scale uses the Likert four-level scoring system, with 0 to 3 points representing non-conformity (0) to very consistent (3). The higher the score, the higher the level of negative emotions.

The authors apologize for this error and state that this does not change the scientific conclusions of the article in any way. The original article has been updated.

## Publisher's Note

All claims expressed in this article are solely those of the authors and do not necessarily represent those of their affiliated organizations, or those of the publisher, the editors and the reviewers. Any product that may be evaluated in this article, or claim that may be made by its manufacturer, is not guaranteed or endorsed by the publisher.
